# Antimicrobial resistance and associated genetic background of *Histophilus somni* isolated from clinically affected and healthy cattle

**DOI:** 10.3389/fvets.2022.1040266

**Published:** 2022-10-25

**Authors:** Yuichi Ueno, Kenta Suzuki, Yuji Takamura, Kaori Hoshinoo, Daisuke Takamatsu, Ken Katsuda

**Affiliations:** ^1^Division of Infectious Animal Disease Research, National Institute of Animal Health, National Agriculture and Food Research Organization, NARO, Tsukuba, Japan; ^2^Nagano Prefectural Matsumoto Livestock Hygiene Service Center, Matsumoto, Japan; ^3^Aichi Prefectural Chuo Livestock Hygiene Service Center, Okazaki, Japan; ^4^The United Graduate School of Veterinary Sciences, Gifu University, Gifu, Japan; ^5^National Institute of Animal Health, National Agriculture and Food Research Organization, NARO, Tsukuba, Japan

**Keywords:** *Histophilus somni*, antimicrobial resistance, bovine respiratory disease, antimicrobial resistance genes, integrative and conjugative elements

## Abstract

*Histophilus somni*, a member of the *Pasteurellaceae* family, causes various diseases, including thrombotic meningoencephalitis and respiratory diseases. Here, 166 isolates recovered from Japanese cattle with various diseases between the late 1970s and the 2010s were subjected to susceptibility testing against 14 antimicrobials (ampicillin, amoxicillin, cefazolin, ceftiofur, kanamycin, streptomycin, nalidixic acid, enrofloxacin, danofloxacin, florfenicol, erythromycin, tylosin, oxytetracycline, and fosfomycin). The proportions of antimicrobial-resistant/intermediate isolates were low in the total isolates, with resistance rates ranging from 0% for ceftiofur and florfenicol to 13.2% for ampicillin. However, relatively high minimum inhibitory concentrations (MICs) and resistance/intermediate rates were observed in the isolates from cattle with respiratory diseases; i.e., 21/53 isolates (39.6%) showed resistance or intermediate to one or more antimicrobials for treatment of respiratory diseases, and the resistance/intermediate rates to oxytetracycline, kanamycin, ampicillin, amoxicillin, nalidixic acid, and danofloxacin were 28.3, 24.5, 24.5, 13.2, 1.9, and 1.9%, respectively. Isolates with high MICs tended to possess antimicrobial resistance genes, which may confer antimicrobial resistance phenotypes. In particular, all isolates with MICs of ampicillin/amoxicillin, kanamycin, and oxytetracycline ≥2 μg/mL, ≥512 μg/mL, and ≥4 μg/mL possessed *bla*_ROB − 1_, *aphA-1*, and *tetH*/*tetR*, respectively, whereas isolates whose MICs were lower than the above-mentioned values did not possess these resistance genes. These results suggest that the resistance genes detected in this study are primarily responsible for the reduced susceptibility of *H. somni* strains to these antimicrobials. As integrative and conjugative element (ICEs)-associated genes were detected only in genetically related isolates possessing antimicrobial resistance genes, ICEs may play an important role in the spread of resistance genes in some genetic groups of *H. somni* strains.

## Introduction

The emergence and spread of antimicrobial-resistant pathogens poses a substantial global threat to human and animal health. In the livestock industry, animals are often raised using large amounts of antimicrobials to prevent and treat infectious diseases as well as for growth promotion (1, 2). The extensive use of antimicrobials in the industry may cause the emergence and selection of antimicrobial-resistant pathogens in livestock. These resistant pathogens may result in treatment failure, which leads to economic losses, and may also be a source of resistant bacteria/genes that present a risk to human health. To reduce antimicrobial resistance (AMR) and preserve the effectiveness of currently available antimicrobials, the World Health Organization (WHO) included five strategic objectives in the global action plan on AMR. Strengthening knowledge of AMR through research to understand its current status and mechanisms of emergence and transmission is an important objective to achieve this goal (3).

*Histophilus somni* is a member of the *Pasteurellaceae* family. Most strains of this bacterium have been grouped into nine genetic clades (Ia, Ib, and II-VIII) based on the major outer membrane protein (MOMP) gene sequences and ten clusters based on the pulsed-field gel electrophoresis (PFGE) profiles (HS1-HS10) (4). This Gram-negative pleomorphic rod is an important bacterial pathogen involved in the multifactorial etiology of bovine respiratory disease (BRD). It also causes septicemia, thrombotic meningoencephalitis (TME), myocarditis, and abortion in cattle and sheep. Although various approaches, including vaccination, antimicrobial treatment, and hygiene management, have been used for disease control, histophilosis remains an important cause of mortality and economic loss in Japan and other countries (4–7).

Antimicrobial therapy is one of the most effective tools for the treatment of BRD caused by *H. somni* and other *Pasteurellaceae* species, i.e., *Pasteurella multocida* and *Mannheimia haemolytica*. However, the excessive and unreasonable use of antimicrobials has accelerated selective pressure, which may affect the expression of antimicrobial resistance genes and enhance the emergence of resistant isolates in these microorganisms. Notably, an increase in the incidence of multidrug-resistant pathogenic bacteria has been reported in recent decades, and is gradually decreasing the efficacy of currently available antibiotics for BRD in North America (8–10). Regardless, there are only a few studies on the current AMR status of *H. somni* compared to other species of the family *Pasteurellaceae* (7, 8, 11–13). In Japan, this bacterium has not been subjected to national surveillance of AMR conducted by the Japanese Veterinary Antimicrobial Resistance Monitoring System, and the AMR status of *H. somni* remains unknown.

Acquisition of AMR genes *via* horizontal gene transfer through conjugation, transformation, or transduction is the main mechanism by which bacteria acquire resistance to antimicrobial agents (14). To date, a number of AMR genes that decrease susceptibility to antimicrobials have been reported in the *Pasteurellaceae* family of veterinary origin, including *H. somni* (15). Plasmids are the main conjugation systems for transmitting AMR genes; however, chromosome-borne mobile genetic elements (MGEs), referred to as integrative and conjugative elements (ICEs), have also been identified in the *Pasteurellaceae* family (9, 16–18). These elements usually harbor cassettes of AMR genes and are propagated through recombination in BRD-associated *Pasteurellaceae* (9, 16–18). Once resistant strains are generated *via* MGEs, the population of AMR strains is expected to increase with the use of antimicrobials owing to selective pressure (8, 19). Therefore, it is important to screen AMR- and ICE-associated genes of *H. somni* strains to predict the future emergence and spread of AMR in this bacterium and to plan appropriate measures to prevent the distribution of resistant strains. However, large-scale screening of these genes in *H*. *somni* has not been conducted.

The objectives of this study were (1) to describe AMR in *H. somni* isolated from Japanese cattle with various diseases, (2) to investigate the relationships between AMR phenotypes and AMR and ICE-associated genes, and (3) to investigate the phyletic relationships of resistant isolates.

## Materials and methods

### Bacterial isolates and culture conditions

Here, regardless of geography, isolation time/year, and disease conditions, we selected one isolate per case from among all *H. somni* isolates recovered at prefectural livestock hygiene service centers between 1978 and 2017 and stored at the National Institute of Animal Health, National Agriculture and Food Research Organization for diagnostic examination or scientific research, and a total of 166 *H. somni* isolates from cattle in Japan, including 56 isolates from TME, 53 from respiratory disease, 10 from other diseases (myocarditis [*n* = 4], mastitis [*n* = 4], abortion [*n* = 1], and endocarditis [*n* = 1]), and 20 from clinically healthy cattle, were used (Table 1 and Supplementary Table S1). No animal samples were directly collected by the authors. The status of the animals from which the other 27 isolates originated is unknown. There was no information on the antimicrobial treatment administered to the animals at the time of bacterial isolation. Of the 166 isolates, 128 were genetically characterized based on MOMP gene sequencing and PFGE analysis in a previous study (4) (Table 1 and Supplementary Table S1).

**Table 1:** Status of animals sampled and genetic groups of isolates based on the major outer membrane protein (MOMP) gene sequence and pulsed-field gel electrophoresis (PFGE) profile.

**MOMP genetic clade**	**PFGE cluster**	**Status of animals sampled**					**Total**
		**Respiratory**	**TME**	**Others^a^**	**Clinically healthy**	**Unknown**	
Ia	HS1	10	41	3	2	4	60
Ib	HS8	7		1			8
	HS10	6	1			2	9
II	HS3	5	2	2			9
	HS4	4					4
	Others			1	4		5
III	Others	1					1
VI	Others	2	2		3	2	9
VII	HS5	4			2		6
	HS6				5		5
	HS9	4					4
	Others	5		1			6
VIII	Others				1		1
Others	Others				1		1
ND	ND	5	10	2	2	19	38
Total		53	56	10	20	27	166

TME; thrombotic meningoencephalitis, ND; no data ^a^ Others contain myocarditis (n = 4), mastitis (n = 4), abortion (n = 1) and endocarditis (n = 1).

All isolates were cultured on brain heart infusion (BHI) agar (Difco; BD, New Jersey, USA) plates supplemented with 5% defibrinated sheep blood and 0.5% yeast extract (Difco; BD) at 37 °C under air plus 5% CO_2_ conditions in a water jacket incubator (SCI-325D, Astec, Fukuoka, Japan) for 16 h for antimicrobial susceptibility tests. All isolates were identified as *H. somni* using an *H. somni*-specific PCR assay (20) (Supplementary Table S2) as well as biochemical tests using established methods (4, 21) or a commercial biochemical identification kit, ID-test-HN-20 Rapid (Nissui Pharmaceutical Co. Ltd., Tokyo, Japan). All isolates were maintained in BHI broth (Difco; BD) containing 20% glycerol at −80°C until use.

### Antimicrobial susceptibility testing

Minimum inhibitory concentration (MIC) values of 14 antimicrobials across eight classes: penicillins [ampicillin (ABPC, FUJIFILM Wako, Osaka, Japan), amoxicillin (AMPC, FUJIFILM Wako)]; cephalosporins [cefazolin (CEZ, FUJIFILM Wako), ceftiofur (CTF, FUJIFILM Wako)]; aminoglycosides [kanamycin (KM, FUJIFILM Wako), streptomycin (SM, FUJIFILM Wako)]; quinolones [nalidixic acid (NA, FUJIFILM Wako), enrofloxacin (ERFX, LKT labs, Minnesota, USA), danofloxacin (DNFX, FUJIFILM Wako)]; phenicols [florfenicol (FFC, Combi-Blocks, California, USA)]; macrorlides [erythromycin (EM, FUJIFILM Wako), tylosin (TS, FUJIFILM Wako)]; tetracyclines [oxytetracycline (OTC, FUJIFILM Wako)]; and another [fosfomycin (FOM, FUJIFILM Wako)] were determined using a broth microdilution method with a 96-well microtiter plate (Violamo, Osaka, Japan) according to standard methods of the Clinical and Laboratory Standards Institute (CLSI) guidelines (22). Among the antimicrobial agents, ABPC, AMPC, KM, SM, and OTC have been recommended as first-choice drugs for the treatment of BRD in Japan. *H. somni* ATCC 700025 and *Actinobacillus pleuropneumoniae* ATCC 27090 were used as quality control strains. The MIC was defined as the lowest antimicrobial concentration that inhibited bacterial growth. When bacterial growth was observed at the highest antimicrobial concentration, the MIC was described as greater than the highest concentration; when no bacterial growth was observed at the lowest concentration, the lowest concentration was described as the MIC. The antimicrobial phenotypes of ABPC, CTF, ERFX, DNFX, FFC, and OTC were interpreted on the basis of breakpoints provided by the CLSI guidelines (23), where *H. somni* or other *Pasteurellaceae* species are available. Among MIC distributions for the eight antimicrobials whose breakpoints were unavailable, those of AMPC, KM, and NA clearly showed a bimodal structure, and isolates distributed in higher MIC ranges were considered resistant in this study. For the other antimicrobials (CEZ, SM, EM, TS, and FOM), MIC_50_ and MIC_90_ were used as indices expressing AMR in this study.

### PCR detection of AMR genes and ICE-associated genes

PCR was conducted to detect 13 resistance genes (ABPC and AMPC resistance genes: *bla*_ROB − 1_ and *bla*_OXA − 2_, KM resistance genes: *aadB* and *aphA-1*, SM resistance genes: *strA, strB*, and *aadA25*, FFC resistance gene: *floR*, EM and TS resistance genes: *erm(42), msrE*, and *mphE*, and OTC resistance genes: *tetH* and *tetR*) (17, 24) and four ICE-associated genes (*parB*, ICE-*rel1, int1*, and *int2*) (18) according to previous studies. Oligonucleotide primers and annealing conditions are listed in Supplementary Table S2. DNA manipulation was performed as described previously (25). For DNA preparation, a few bacterial colonies were transferred from an agar plate with a sterile inoculating loop to 500 μL of distilled water and boiled for 10 min. Following centrifugation (12,000 × *g* for 2 min) using an Eppendorf centrifuge 5457 C (Eppendorf, Hamburg, Germany), 1 μL of the supernatant was used as the DNA template for PCR amplification. PCR was performed on a GeneAmp PCR System 9700 (Applied Biosystems, Foster City, CA, USA) using KOD-plus polymerase (TOYOBO, Osaka, Japan) for resistance gene detection or the QIAGEN^®^ Multiplex PCR kit (QIAGEN, Hilden, Germany) for ICE-associated gene detection. Randomly selected PCR products were sequenced to determine the specificity of amplification.

### β-lactamase detection

When resistance or intermediate resistance to at least one of the four antimicrobials (ABPC, AMPC, CEZ, and CTF) was observed, β-lactamase production was examined using a cephinase disc (BBL; BD) regardless of the presence or absence of *bla* genes. *Staphylococcus aureus* ATCC 29213 was used as the positive control. Representative strains of various genetic groups that did not show resistance to the four antimicrobials were also examined for β-lactamase productivity as negative controls in this study.

### Statistical analysis

The association between AMR phenotypes and possessions of associated AMR gene(s) was analyzed using the Fisher's exact test when the associated AMR gene(s) were detected. All statistical analyses were performed with EZR software ver.1.55 (Saitama Medical Center, Jichi Medical University, Saitama, Japan). The association was considered significant at *P* < 0.01.

## Results and discussion

### AMR situation of *H. somni* in Japan

The MIC distributions of 166 *H. somni* isolates, the percentage of resistance and intermediate resistance in each antimicrobial, as well as the MIC_50_ and MIC_90_ values are summarized in Table 2. The background information and MIC values for all isolates are shown in Supplementary Table S1. The MICs of reference strains in each test run were within acceptable CLSI quality control ranges. Overall, 35 of 166 isolates were resistant or intermediate resistant to one or more antimicrobials with seven resistance patterns (ABPC, OTC, KM-OTC, NA-DNFX, NA-ERFX-DNFX, ABPC-AMPC-KM-OTC, and ABPC-AMPC-KM-NA-DNFX-OTC). The number and proportion of resistant and intermediate-resistant isolates were follows: ABPC (22/166, 13.2%), AMPC (7/166, 4.2%), KM (16/166, 9.6%), NA (3/166, 1.8%), ERFX (1/166, 0.6%), DNFX (3/166, 1.8%), and OTC (18/166, 10.8%). Neither resistant nor intermediate-resistant isolates were observed in the CTF and FFC. In addition, differences between MIC_50_ and MIC_90_ of the other antimicrobials (CEZ, SM, EM, TS, and FOM) were within 4-fold (Table 2), suggesting that all isolates were susceptible to the antimicrobials. The MIC ranges and MIC_50_ values in this study were largely similar to those of previous studies, in which the same antimicrobials were used (8, 13, 26–28).

**Table 2:** Minimum inhibitory concentration (MIC) distribution frequencies of *Histophilus somni* isolates.

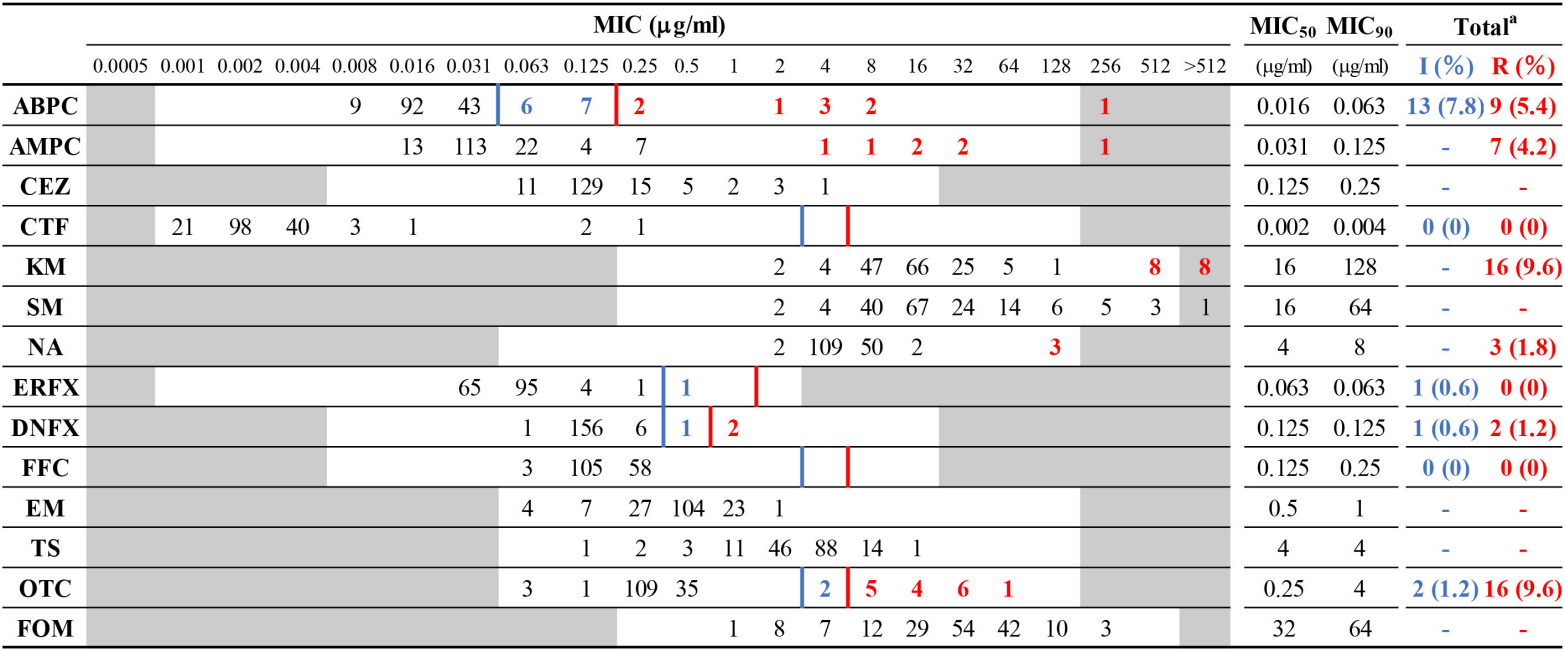

The distribution range of each antimicrobial in this study was those contained in the white area. Values above this range indicate MIC values higher than the highest concentrations within that range. Susceptible and resistant breakpoints are indicated by vertical blue and red lines, respectively, when the CLSI criteria available (23). The breakpoints of DNFX were Pasteurella multocida and Mannheimia haemolytica (23). The numbers in red indicate the isolation number of resistant isolates, blue indicates those of intermediate isolates, and black indicates those of susceptible isolates. ABPC, ampicillin; AMPC, amoxicillin; CEZ, cefazolin; CTF, ceftiofur; KM, kanamycin; SM, streptomycin; NA, nalidixic acid; ERFX, enrofloxacin; DNFX, danofloxacin; FFC, florfenicol; EM, erythromycin; TS, tylosin; OTC, oxytetracycline; FOM, fosfomycin -, undefined ^a^Total number and proportion of intermediate (I) and resistant (R) isolates.

The relationships between the resistance phenotypes and the status of the animals sampled are summarized in Table 3 and detailed in Supplementary Table S3. Although the proportions of resistant and intermediate-resistant isolates were generally low, as described above, isolates from respiratory diseases tended to show higher resistance and intermediate resistance rates than those from other clinical manifestations; i.e., 21 out of 53 isolates from respiratory diseases showed resistance or intermediate resistance to one or more antimicrobials (resistance rate: 39.6%). In addition, the MIC_90_ of ABPC, AMPC, CEZ, KM, SM, and OTC of respiratory disease isolates were 4- to 256-fold higher than that of the isolates from the other clinical statuses (Table 3). Alternatively, differences in the MIC_90_ of the other antimicrobials between respiratory isolates and the rest were within 2-fold. Among antimicrobials, tetracyclines (56,882.5 kg/year), penicillins (14,610.3 kg/year), and aminoglycosides (4520.7 kg/year) are the primary antimicrobials used in the cattle industry in Japan (29). Relatively high resistance rates were observed in respiratory disease isolates to these antimicrobials. In contrast, CTF, NA, ERFX, DNFX, FFC, EM, TS, and FOM seem to have maintained high efficacy for the treatment of BRD caused by *H. somni* in Japan, considering the low number of resistant isolates to and/or low MICs of these antimicrobials.

**Table 3:** Relationships between status of animals sampled and antimicrobial resistance.

	**Status of animals sampled**																			
	**Respiratory (*****n*** = **53)**				**TME** ***(n*** = **56)**				**Others** ^a^ **(*****n*** = **10)**				**Clinically healthy (*****n*** = **20)**				**Unknown (n** = **27)**			
**Antimicrobial agents**	**MIC**_50_	**MIC**_90_	**resistance** ^b^		**MIC**_50_	**MIC**_90_	**resistance** ^b^		**MIC**_50_	**MIC**_90_	**resistance** ^b^		**MIC**_50_	**MIC**_90_	**resistance** ^b^		**MIC**_50_	**MIC**_90_	**resistance** ^b^	
	**(mg/ml)**	**(mg/ml)**	**I**	**R**	**(mg/ml)**	**(mg/ml)**	**I**	**R**	**(mg/ml)**	**(mg/ml)**	**I**	**R**	**(mg/ml)**	**(mg/ml)**	**I**	**R**	**(mg/ml)**	**(mg/ml)**	**I**	**R**
ABPC	0.031	4	6 (11.3)	7 (13.2)	0.0156	0.063	5 (8.9)	1 (1.8)	0.0156	0.031	0 (0)	1 (10)	0.0156	0.156	0 (0)	0 (0)	0.0156	0.031	2 (7.4)	0 (0)
AMPC	0.031	8	-	7 (13.2)	0.031	0.063	-	0 (0)	0.031	0.063	-	0 (0)	0.031	0.031	-	0 (0)	0.031	0.063	-	0 (0)
CEZ	0.125	1	-	-	0.125	0.25	-	-	0.125	0.125	-	-	0.125	0.125	-	-	0.125	0.25	-	-
CTF	0.002	0.004	0 (0)	0 (0)	0.002	0.004	0 (0)	0 (0)	0.002	0.004	0 (0)	0 (0)	0.002	0.002	0 (0)	0 (0)	0.002	0.004	0 (0)	0 (0)
KM	32	512	-	13 (24.5)	16	32	-	1 (1.8)	16	32	-	0 (0)	8	16	-	0 (0)	16	64	-	2 (7.4)
SM	32	256	-	-	16	64	-	-	16	32	-	-	8	16	-	-	16	64	-	-
NA	4	8	-	1 (1.9)	4	8	-	1 (1.8)	4	8	-	1 (10)	4	8	-	0 (0)	4	8	-	0 (0)
ERFX	0.063	0.063	0 (0)	0 (0)	0.063	0.063	0 (0)	0 (0)	0.063	0.063	0 (0)	0 (0)	0.031	0.063	0 (0)	0 (0)	0.063	0.063	0 (0)	0 (0)
DNFX	0.125	0.125	1 (1.9)	0 (0)	0.125	0.125	0 (0)	1 (1.8)	0.125	0.125	0 (0)	1 (10)	0.125	0.125	0 (0)	0 (0)	0.125	0.125	0 (0)	0 (0)
FFC	0.125	0.25	0 (0)	0 (0)	0.125	0.25	0 (0)	0 (0)	0.125	0.25	0 (0)	0 (0)	0.125	0.25	0 (0)	0 (0)	0.125	0.25	0 (0)	0 (0)
EM	0.5	1	-	-	0.5	0.5	-	-	0.5	1	-	-	0.5	0.5	-	-	0.5	1	-	-
TS	4	8	-	-	4	4	-	-	4	8	-	-	2	4	-	-	4	4	-	-
OTC	0.25	16	2 (3.8)	13 (24.5)	0.5	1	0 (0)	1 (1.8)	0.25	0.5	0 (0)	0 (0)	0.25	0.5	0 (0)	0 (0)	0.25	0.5	0 (0)	2 (7.4)
FOM	32	64	-	-	32	64	-	-	32	64	-	-	16	64	-	-	32	128	-	-

TME, thrombotic meningoencephalitis; ND, no data, -, undefined.

The numbers in red indicate the isolation number and proportion of resistant isolates, blue indicates those of intermediate isolates.

a Others included myocarditis (n = 4), mastitis (n = 4), abortion (n = 1), and endocarditis (n = 1).

b Total number and proportion (%) of intermediate (I) and resistant (R) isolates.

**Figure 1:**
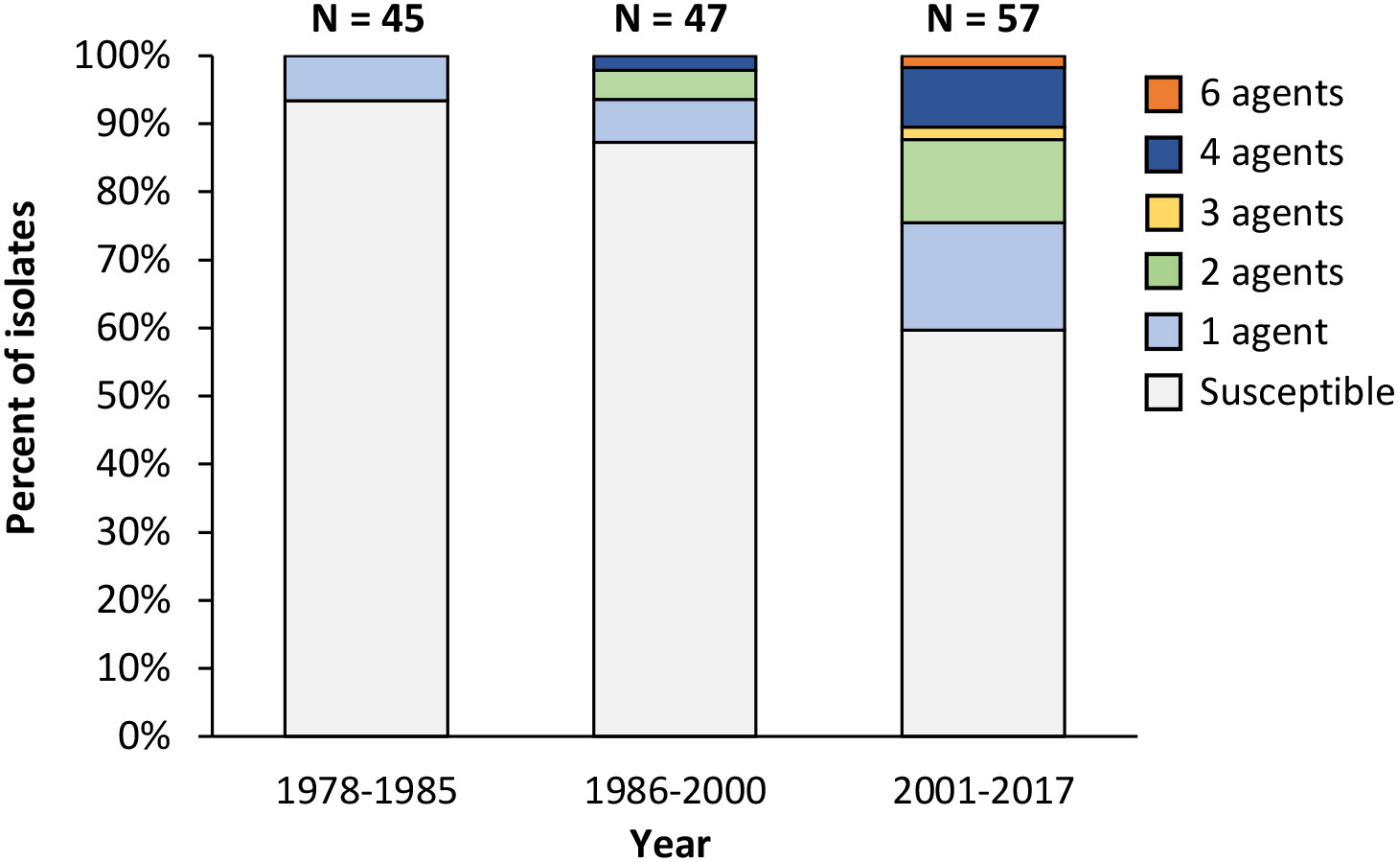
Trends in the antimicrobial resistance pattern of *Histophilus somni*. Isolates with information on the isolation years (*n* = 149) are used in this figure. Susceptible: Susceptible to all antimicrobial agents used in this study; 1 agent−4 agents, 6 agents: resistant to the indicated number of antimicrobial agents.

Most of the resistant isolates were isolated after 2000 (Table 4), and the resistance rate has increased over time (Figure 1). This is probably because the number of *H. somni* isolates from respiratory diseases has increased since the late 1990s when the bacterium was recognized as an important causative agent of respiratory diseases in Japan (4). There has been no remarkable change in the sales amount of antimicrobials for animal use in Japan since 1998, when the sales amount data became available, and the reason for the decreased susceptibility observed in the present study is unknown. In Japan, appropriate use of antimicrobials in the field of livestock production has been promoted especially since 2016, when the national action plan on AMR in Japan was launched. Continuous AMR investigation will reveal the influence of the promotion on AMR of *H. somni*.

**Table 4:** Information of isolates that showed resistance to one or more antimicrobials.

**ID**	**Strain**	**Prefecture**	**Year**	**Status of**	**Anatomical**	**MOMP**	**PFGE**	**Resistance pattern**	**Resistance gene**	**ICE-associated gene**	**Reference**
				**animal**		**genetic**	**cluster**				**or source**
						**clade**					
**One or more resistant gene and ICE-asseciated gene were detected**											
BD113	GM-29	Gunma	1993	TME	Brain	Ib	HS10	KM, OTC	*aphA-1, tetH, tetR*	ICE*-rel1, int1, int2*	(4)
BD118	GM-34	Gunma	1995	Pneumonia	Lung	Ib	HS8	ABPC, AMPC, KM, OTC	*bla*_ROB − 1_ *, aphA-1, strA, strB, tetH, tetR*	ICE*-rel1, int1, int2*	(4)
BD130	GM-46	Gunma	2000	Pneumonia	Lung	Ib	HS8	KM, OTC	*aphA-1, tetH, tetR*	ICE*-rel1, int1, int2*	(4)
BD132	GM-48	Gunma	2005	Pneumonia	Lung	Ia	HS1	ABPC, AMPC, KM, NA, DNFX, OTC	*bla*_ROB − 1_ *,aphA-1, strA, strB, tetH, tetR*	*parB*, ICE-*rel1, int1, int2*	(4)
BD144	AM-4(745)	Aomori	2008	Respiratory	Nasal	Ib	HS8	OTC	*tetH, tetR*	ICE*-rel1, int1, int2*	(4)
BD650						ND	ND	KM, OTC	*aphA-1, tetH, tetR*	ICE*-rel1, int1, int2*	This work
BD699	FO-5B14(FO-1)	Fukuoka	2004	Pneumonia		VII	HS9	KM, OTC	*aphA-1, strA, strB, tetH, tetR*	*parB*, ICE-*rel1, int1, int2*	(4)
BD708	SAG17-16(SAG-1)	Saga	2005	Pneumonia	Lung	VII	HS9	ABPC, AMPC, KM, OTC	*bla*_ROB − 1_ *, aphA-1, strA, strB, tetH, tetR*	*parB*, ICE-*rel1, int1, int2*	(4)
BD711	KM-1#1(KM-1)	Kumamoto	2005	Pneumonia	Lung	VII	HS9	ABPC, AMPC, KM, OTC	*bla*_ROB − 1_ *, aphA-1, strA, strB, tetH, tetR*	*parB*, ICE-*rel1, int1, int2*	(4)
BD1294	N47	Yamaguchi	2001	Respiratory	Nasal	Ib	HS8	ABPC, AMPC, KM, OTC	*bla*_ROB − 1_ *, aphA-1, strA, strB, tetH, tetR*	*parB*, ICE-*rel1, int1, int2*	(4)
BD1296	N73	Yamaguchi	2002	Respiratory	Nasal	Ib	HS8	ABPC, AMPC, KM, OTC	*bla*_ROB − 1_ *, aphA-1, strA, strB, tetH, tetR*	*parB*, ICE-*rel1, int1, int2*	(4)
BD1310	M1397	Yamaguchi	2010	Pneumonia	Lung	Ia	HS1	KM, (OTC)	*aphA-1, strA, strB, tetH, tetR*	*parB*, ICE-*rel1, int1, int2*	(4)
BD1311	D175	Yamaguchi	2011		Lung	Ia	HS1	KM, OTC	*aphA-1, strA, strB, tetH, tetR*	*parB*, ICE-*rel1, int1, int2*	(4)
BD1317	KM-3 original	Kumamoto	2005	Pneumonia	Lung	VII	HS9	ABPC, AMPC, KM, OTC	*bla*_ROB − 1_ *, aphA-1, strA, strB, tetH, tetR*	*parB*, ICE-*rel1, int1, int2*	(4)
BD1318	NGS-1	Nagasaki	2015	Pneumonia	Lung	Ib	HS10	(OTC)	*tetH, tetR*	ICE*-rel1, int1, int2*	(4)
BD1345	YGT17-1	Yamagata	2005	Pneumonia	Lung	Ib	HS10	KM, OTC	*aphA-1, tetH, tetR*	ICE*-rel1, int1, int2*	(4)
BD1348	YGT18-102	Yamagata	2006	Respiratory	Nasal	Ib	HS10	KM, OTC	*aphA-1, strA, strB, tetH, tetR*	*parB*, ICE-*rel1, int1, int2*	(4)
BD1350	YGT20-130	Yamagata	2008	Pneumonia	Lung	Ib	HS10	KM, OTC	*aphA-1, tetH, tetR*	ICE*-rel1, int1, int2*	(4)
**No Resistant gene and ICE-asseciated gene were detected**											
BD21	NN-12	Nagano	1987	Pneumonia		VII	Others	(ABPC)	-	-	(4)
BD79	HS20-1	Hiroshima	1992	TME	Brain	Ia	HS1	(ABPC)	-	-	(4)
BD103	IK-13	Ibaraki	2001	Pneumonia		VII	Others	(ABPC)	-	-	(4)
BD114	GM-30	Gunma	1993	Pneumonia	Lung	VII	Others	(ABPC)	-	-	(4)
BD136	GM-52	Gunma	2006	TME	Brain	II	HS3	NA, ERFX, DNFX	-	-	(4)
BD143	AM-3(744)	Aomori	2008	Respiratory	Nasal	Ia	HS1	(ABPC)	-	-	(4)
BD293	GM638-08-637	Gunma	2009	TME	Heart	Ia	HS1	(ABPC)	-	-	(4)
BD294	GM743-10-94	Gunma	2010	TME	Brain	Ia	HS1	(ABPC)	-	-	(4)
BD316	HD-10	Hokkaido	Before 1982	TME		Ia	HS1	(ABPC)	-	-	(4)
BD318	HD-12	Hokkaido	Before 1982	TME		Ia	HS1	ABPC	-	-	(4)
BD608						ND	ND	(ABPC)	-	-	This work
BD630						ND	ND	(ABPC)	-	-	This work
BD660			1981	TME		ND	ND	(ABPC)	-	-	This work
BD699	FO-5B14(FO-1)	Fukuoka	2004	Pneumonia		VII	HS9	(ABPC)	-	-	(4)
BD701	FO-6D4(FO-3)	Fukuoka	2006	Myocarditis	Heart	II	HS3	NA, DNFX	-	-	(4)
BD1314	D995	Yamaguchi	2015	Mastitis	Milk	VII	Others	ABPC	-	-	(4)
BD1354	YGT27-87	Yamagata	2015	Pneumonia	Lung	II	HS4	(ABPC)	-	-	(4)
The other 131 isolates								Not resistant to any antimicrobials	-	-	

ND, no data; -, not detected.

Antimicrobials in parenthesises means intermediate resistance.

Resistance rates, MIC_50_, and MIC_90_ in isolates from TME were lower than those from respiratory diseases, and no resistance was observed in isolates from clinically healthy cattle in this study (Table 3). The reason for the high susceptibility is probably that cattle with acutely progressive TME and clinically healthy cattle have fewer opportunities for antimicrobial treatment than those with respiratory diseases. According to a previous study on antimicrobial susceptibility of *H. somni* isolated from cattle with TME in Japan, in which ABPC, KM, SM, NA, EM, TS, and OTC were used, four of the 45 isolates showed resistance to SM only (30). In the present study, although six ABPC resistant or intermediate resistant, one KM-OTC resistant, and one NA-ERFX-DNFX-resistant isolates from TME were newly detected (Table 4), no notable change was observed in the AMR situation of TME isolates in Japan. Among the other 10 isolates from clinically affected cattle, four from myocarditis, four from mastitis, one from abortion, and the other from endocarditis, one isolate from myocarditis showed NA-DNFX resistance and another from mastitis showed ABPC resistance (Tables 3, 4). More isolates need to be used to analyze the relationship between AMR and these diseases in cattle.

### AMR genes and ICE-associated genes were found in isolates that showed resistance to one or more antimicrobials

In this study, we demonstrated the relationships between AMR phenotypes and associated genetic components, AMR genes, and ICE-associated genes in *H. somni* isolates. The genetic components detected in this study are summarized in Table 4. Among the 13 AMR genes and four ICE-associated genes screened, six AMR genes (*bla*_ROB − 1_, *aphA-1, strA, strB, tetH*, and *tetR*) and all four ICE-associated genes (*parB*, ICE-*rel1, int1*, and *int2*) were detected among the 18 isolates that showed resistance or intermediate resistance to one or more antimicrobials used in this study (Table 4).

The presence of AMR genes was significantly (*P* < 0.0001) related to the associated AMR phenotypes (Table 5). In particular, all seven isolates with MICs of ABPC and AMPC ≥2 μg/mL and ≥4 μg/mL, respectively, possessed a β-lactamase coding gene, *bla*_ROB − 1_ (Table 5), and β-lactamase production of the isolates was confirmed using a cephinase disc (BBL; BD) (Supplementary Table S1). To our knowledge, this is the first study to detect *bla*_ROB − 1_ in *H. somni* isolates. In contrast, *bla*_ROB − 1_ was not detected in the other 159 isolates, including 15 isolates that showed resistance or intermediate resistance to ABPC (Table 4). However, MIC ranges of the 15 isolates to ABPC were clearly low (0.063–0.25 μg/mL) compared with those of the other seven *bla*_ROB −1_-positive ABPC resistant isolates (2–>256 μg/mL). Furthermore, *bla*_ROB −1_ was present in all AMPC-resistant isolates with MICs of AMPC ≥4 μg/mL. These results strongly suggested that *bla*_ROB − 1_ is responsible for the remarkable decrease in the susceptibility of *H. somni* to ABPC and AMPC. In *Haemophilus influenzae*, some amino acid substitutions in the *bla*_ROB − 1_ gene are known to confer resistance to penicillins and cephalosporins (31). However, in the present study, the seven *bla*_ROB − 1_-positive isolates were susceptible to CEZ and CTF, suggesting that the *bla*_ROB − 1_ gene in *H. somni* isolates is not equipped with the ability to confer resistance to cephalosporins. All 16 KM-resistant (MIC ≥512 μg/mL) and 18 OTC-resistant or intermediate resistant (MIC ≥4 μg/mL) isolates possessed the associated resistance genes *aphA-1* and *tetH*/*tetR*, respectively (Table 5). Eleven of the 16 SM-resistant isolates with MICs ≥128 μg/mL possessed *strA* and *strB*. Considering these results, *strA* and *strB* may decrease susceptibility to SM, and the breakpoint of *H. somni* for SM could be set to approximately 128 μg/mL. However, none of the isolates susceptible to these antimicrobials possessed the AMR genes (Table 5). These findings suggested that the resistance of Japanese *H. somni* isolates to KM, OTC, ABPC, and AMPC may be associated with one or two resistance genes. Among the relationships between AMR phenotypes and associated AMR genes that we found, those of OTC resistance and *tetH*/*tetR* has also been suggested in previous studies on AMR in *H. somni* (32, 33), while the other relationships (i.e., those between ABPC/AMPC resistance and *bla*_ROB−1_, KM resistance and *aphA-1*, and SM resistance and *strA*/*strB* in *H. somni*) were suggested for the first time in the present study. However, the presence of the genes does not indicate its expression and function. The association between AMR phenotypes and associated AMR genes should be concluded after confirmation on the gene expression and function. AMR-associated genes, *bla*_OXA−2_, *aadB* and *aadA25* were not detected in the isolates regardless of their resistance or susceptibility to associated antimicrobials. The other AMR genes, *floR, erm (42), msrE*, and *mphE* were also not detected, which is consistent with the results that all isolates were susceptible to FFC, EM, and TS, respectively.

**Table 5:** Fisher's exact test between antimicrobial resistance (AMR) phenotypes and AMR genes.

**Ampicillin**		**PCR**	
		***bla*** **_ROB − 1_ +**	***bla*** **_ROB − 1_ *-***
MIC (mg/ml)	≥2	7	0
	< 2	0	159

The two-tailed P-value is < 0.0001.

+, positive; -, negative.

**Table:** 

**Amoxicillin**		**PCR**	
		***bla*** **_ROB − 1_ +**	***bla*** **_ROB − 1_ *-***
MIC (mg/ml)	≥4	7	0
	<4	0	159

The two-tailed P-value is < 0.0001.

+, positive; -, negative.

**Table:** 

**Kanamycin**		**PCR**	
		***aphA1*** **+**	***aphA1*** **-**
MIC (mg/ml)	≥512	16	0
	< 512	0	150

The two-tailed P-value is < 0.0001.

+, positive; -, negative.

**Table:** 

**Streptomycin**		**PCR**	
		***strA+, strB+***	***strA-, strB-***
MIC (mg/ml)	≥128	11	5
	<128	0	150

The two-tailed P-value is < 0.0001.

+, positive; -, negative.

**Table:** 

**Oxytetracycline**		**PCR**	
		***tetH*** **+, *tetR*+**	***tetH*** **-, *tetR*-**
MIC (mg/ml)	≥4	18	0
	<4	0	148

The two-tailed P-value is < 0.0001.

+, positive; -, negative.

In *H. somni*, the presence of ICE-associated genes has been reported to have less of an effect on MICs than those in other *Pasteurellaceae, P. multocida*, and *M. haemolytica* (33). In the present study, three of the four ICE-associated genes were exclusively present in 18 isolates carrying the AMR genes investigated (Table 4). In particular, the tetracycline resistance gene *tetH* and its repressor gene *tetR* were detected in all 18 isolates. Previous studies have also reported the presence of ICE associated with *tetH* in *Pasteurellaceae* (9, 33–35). Other AMR genes were also detected among the isolates: *bla*_ROB − 1_, seven isolates; *aphA-1*,16 isolates; and *strA* and *strB*,11 isolates. These AMR genes are reported to be present on the ICE in *H. somni* and *P. multocida*, with the exception of *bla*_ROB − 1_, and all genes, including *bla*_ROB − 1_, are reported to be present on the ICE in *M. haemolytica* (9, 33, 34). In contrast, no ICE-associated genes were detected in the 148 isolates that did not possess any AMR genes screened in this study. These findings suggested that AMR genes may be carried on ICEs in Japanese isolates as well, although wide-range sequencing analyses of ICE-associated genes are required to confirm this hypothesis.

### AMR genes and ICE-associated genes were present only in the isolates assigned to the four genetic groups

Among the 166 isolates used in this study, 128 isolates were genetically analyzed in a previous study (4) and grouped into seven MOMP genetic clades (clades Ia, Ib, II, III, and VI–VIII) or others and eight PFGE clusters (HS1, HS3–HS6, and HS8–HS10) or others. Based on the combinations of MOMP genetic clades and PFGE clusters, *H. somni* isolates used in this study were grouped into at least 14 genetic groups (Table 1). In the present study, except for one isolate that had no genotyping data, all isolates that carried both AMR genes and ICE-associated genes were grouped into four genetic groups (Ia-HS1: three isolates, Ib-HS8: five isolates, Ib-HS10: five isolates, VII-HS9: four isolates). In addition, the AMR and ICE-associated gene-positive isolates contained at least 15 isolates from BRD cases (Table 4). The proportion of isolates that carried both AMR- and ICE-associated genes in each genetic group was 5.0% (3/60 isolates) in Ia-HS1, 62.5 % (5/8 isolates) in Ib-HS8, 55.6% (5/9 isolates) in Ib-HS10, and 100% (4/4 isolates) in VII-HS9. Although our results suggested that AMR genes may have been maintained *via* ICEs in genetically limited *H. somni* populations, especially those on the respiratory mucous membrane, further analyses are needed to elucidate the relationship between AMR and ICEs.

## Conclusions

The present study reports the AMR situation and genetic background of *H. somni* in Japan. This information will be useful for veterinarians and farm managers in selecting effective antimicrobials for the treatment of histophilosis. In particular, the fact that a relatively high percentage of AMR was observed in isolates from respiratory diseases indicates the necessity to select appropriate antimicrobials for the disease to slow the emergence and spread of AMR and to maintain the efficacy of currently available antimicrobials at cattle production sites. Resistance to ABPC, AMPC, KM, and OTC was completely related to the presence of one or two AMR genes and three or four of the four ICE-associated genes. Furthermore, these genes have been found in genetically limited isolates. Although further analysis is needed to elucidate the relationships among AMR phenotypes, AMR genes, and ICE-associated genes, detection of these AMR genes or genotyping may help to rapidly distinguish resistant isolates and to select appropriate antimicrobials for the treatment of bovine histophilosis.

## Data availability statement

The original contributions presented in the study are included in the article/Supplementary materials, further inquiries can be directed to the corresponding authors.

## Author contributions

YU and KK designed the study. YU, KS, and YT performed experiments. YU, KH, DT, and KK analyzed the data. YU and KH contributed to the materials. All authors contributed to the preparation of the manuscript and approved the final version.

## Funding

This study was conducted under the research project on Regulatory research projects for food safety, animal health, and plant protection (grant JPJ008617. 22682153) funded by the Ministry of Agriculture, Forestry, and Fisheries of Japan.

## Conflict of interest

The authors declare that the research was conducted in the absence of any commercial or financial relationships that could be construed as a potential conflict of interest.

## Publisher's note

All claims expressed in this article are solely those of the authors and do not necessarily represent those of their affiliated organizations, or those of the publisher, the editors and the reviewers. Any product that may be evaluated in this article, or claim that may be made by its manufacturer, is not guaranteed or endorsed by the publisher.
